# Morpho-molecular signal correlation between optical coherence tomography and Raman spectroscopy for superior image interpretation and clinical diagnosis

**DOI:** 10.1038/s41598-021-89188-2

**Published:** 2021-05-11

**Authors:** Iwan W. Schie, Fabian Placzek, Florian Knorr, Eliana Cordero, Lara M. Wurster, Gregers G. Hermann, Karin Mogensen, Thomas Hasselager, Wolfgang Drexler, Jürgen Popp, Rainer A. Leitgeb

**Affiliations:** 1 Leibniz Institute of Photonic Technology (Leibniz-IPHT), Albert-Einstein-Straße 9, Jena, 07745 Germany; 2 Department of Medical Engineering and Biotechnology, University of Applied Sciences-Jena, Carl-Zeiss-Promenade 2, 07745 Jena, Germany; 3 Center for Medical Physics and Biomedical Engineering, Medical University of Vienna, Waehringer Guertel 18-20 / 4L, 1090 Vienna, Austria; 4 Department of Urology, Copenhagen University, Herlev/Gentofte Hospital, Borgmester Ib Juuls Vej 23A, 2730 Herlev/Copenhagen, Denmark; 5 Department of Pathology, Copenhagen University, Herlev/Gentofte Hospital, Borgmester Ib Juuls Vej 23A, 2730 Herlev/Copenhagen, Denmark; 6 Institute of Physical Chemistry, Friedrich Schiller University Jena, Helmholtzweg 4, 07743 Jena, Germany

**Keywords:** Biophotonics, Diagnostic markers

## Abstract

The combination of manifold optical imaging modalities resulting in multimodal optical systems allows to discover a larger number of biomarkers than using a single modality. The goal of multimodal imaging systems is to increase the diagnostic performance through the combination of complementary modalities, e.g. optical coherence tomography (OCT) and Raman spectroscopy (RS). The physical signal origins of OCT and RS are distinctly different, i.e. in OCT it is elastic back scattering of photons, due to a change in refractive index, while in RS it is the inelastic scattering between photons and molecules. Despite those diverse characteristics both modalities are also linked via scattering properties and molecular composition of tissue. Here, we investigate for the first time the relation of co-registered OCT and RS signals of human bladder tissue, to demonstrate that the signals of these complementary modalities are inherently intertwined, enabling a direct but more importantly improved interpretation and better understanding of the other modality. This work demonstrates that the benefit for using two complementary imaging approaches is, not only the increased diagnostic value, but the increased information and better understanding of the signal origins of both modalities. This evaluation confirms the advantages for using multimodal imaging systems and also paves the way for significant further improved understanding and clinically interpretation of both modalities in the future.

## Introduction

In the last three decades, biomedical imaging has largely focused on the development and translation of single-modality techniques, e.g. optical coherence tomography (OCT), Raman spectroscopy (RS), fluorescence microscopy, etc. for in-vivo tissue characterization in order to improve medical diagnostics^1^. An individual analytical modality, however, only provides a certain, and usually limited aspect about a sample related to the specific contrast mechanism, potentially missing other relevant diagnostic biomarkers^2^. Consequently, the combination of multimodal approaches was and still is a prime focus in the biomedical optics research. Multimodal approaches are based on two (or more) complementary modalities, covering largely independent biomarker information, and providing a more complete diagnostic evaluation. Especially the combination of modalities that provide morphological and molecular information, such as optical coherence tomography (OCT) and Raman spectroscopy (RS) has been proven to be potentially very powerful^3–5^. OCT, as a non-invasive interferometric-based imaging modality, provides morphologically depth resolved tissue information^6^ with numerous applications in ophthalmology. Furthermore, OCT has also recently been translated to endoscopic applications in cardiovascular, gastrointestinal and urinary tract as well as pulmonary diagnosis^7–9^. While OCT delivers structural information in real time, it falls short in providing molecular or metabolic information of the investigated tissue. Similar to medical ultrasonography the diagnostic evaluation of OCT significantly relies on the expertise of the user and a time-consuming correlation to conventional histological thin section staining methods, such as hematoxylin and eosin (H&E). Raman spectroscopy, on the other hand, is based on inelastic scattering events between photons and molecules, providing non-invasive label-free information on the molecular content of a sample. It has been widely used for single cell^10^ or tissue characterization^11–13^ and a variety of in-vivo applications, inter alia, discrimination of brain tumors^14,15^ or breast tissue^16,17^. A number of implementations also incorporated RS into fiber optical probes for assessment of pathologies for a variety of inner organs^18^. It is, therefore, not surprising that considerable research effort has gone into the multimodal combination of optical coherence tomography and Raman spectroscopy (OCT-RS), which has been applied in various ex-vivo and in-vivo studies^4,5,19–27^. The comprehensive work excellently outlines the advantages for combining those two very complementary optical modalities. It is quite apparent that the physical signal origin for both modalities is different, i.e. in OCT it is the elastic back scattering of photons, due to a change in the refractive index, while in RS, it is the inelastic scattering between photons and molecules. Nevertheless, despite those different characteristics there is a link between both modalities, namely the OCT signal strength is proportional to the scattering property of the tissue, while the scattering property of the tissue is linked to the molecular composition^28^. To be more precise, the scattering intensity will, for example, significantly differ between lipid tissue and fibrous tissue, but so will also the molecular composition for those tissues. While these considerations will only be valid for a well-defined set of samples, e.g. certain biological tissues, it should be possible to relate signals between both modalities, enabling a better signal interpretation of each modality.

In general, many different hollow internal organs of the human body consist of characteristic layers: an epithelial layer, a connective tissue layer and a muscular layer. The epithelial layer covers the organs towards the inner lumen (colon, urinary bladder, etc.); connective tissue is comprised of collagen fibers, nerves, blood and lymphatic vessels^29^; and the muscular layer provides structural integrity of the organ and enables voluntary and involuntary contractile functions, such as heart contraction or movement of the gastrointestinal and urinary tracts. The molecular tissue composition of each layer is diverse, but can be assessed by Raman spectroscopy, through the assessment of distribution of cells, collagen or lipids^30^. Because conventional RS provides no depth perception of the component distribution, the implementation of OCT can compensate this by providing the layered structures of the organ wall as well as transition zones of chemically different composed tissue locations, due to the change in the refractive index between the layers. The urinary bladder wall, for example, consists of the urothelium or mucosa, placed on a connective tissue layer called lamina propria and the muscularis propria^29^. Performing OCT measurements on the inner bladder wall, will result in reduced signal intensity coming from the urothelium and the muscularis layer^31^, while connective tissue, on the other hand, consisting among other components of collagen fibers, will lead to higher OCT signals originating from this layer^32–34^. In contrast, deposits of fatty tissue could result in very low OCT signal, as reported in breast tumor imaging^35–37^. Those OCT signal intensity relations can be quite ambiguous, because OCT signal contrast may have a multitude of different origins, which are not directly deducible from the data. For example, mechanical damage to the tissue caused by surgical equipment may result in low OCT signal areas^38^ and could lead to false interpretation of presented OCT images. Hence, it is paramount to compare OCT data from biopsies to pathological H&E stained thin sections to establish a ground truth for the observation. This, however, also bears significant challenges for ex-vivo analysis and is inconceivable for a co-registration of in-vivo measurements and the extracted biopsy. The combination with a molecular specific modality, which can access the underlying molecular origin and directly correlate the information to the pathological information, would be highly valuable. The ability of Raman spectroscopy to provide molecular information of biological sample that can directly be correlated to H&E information has been extensively shown^39^. However, as of now, the multimodal combination of RS and OCT has largely focused on the extraction of individual biomarkers for disease diagnostics, and not to assess the relation between the two signals, which can considerably increase the understanding for underlying origin of the generated signals.

Here, we investigate for the first time the relation of co-registered OCT and RS signals of human bladder tissue, to demonstrate that the signals of these complementary modalities are inherently intertwined, enabling a direct but more importantly improved interpretation and better comprehension of the other modality. This work demonstrates that the benefit for using two complementary imaging approaches is not only the increased diagnostic value, but the increased information and better understanding of the signal origins of both modalities. This evaluation confirms the advantages for using multimodal imaging systems and also paves the way for significant further improved understanding and clinically interpretation of both modalities in the future.

## Results

**Figure 1:**
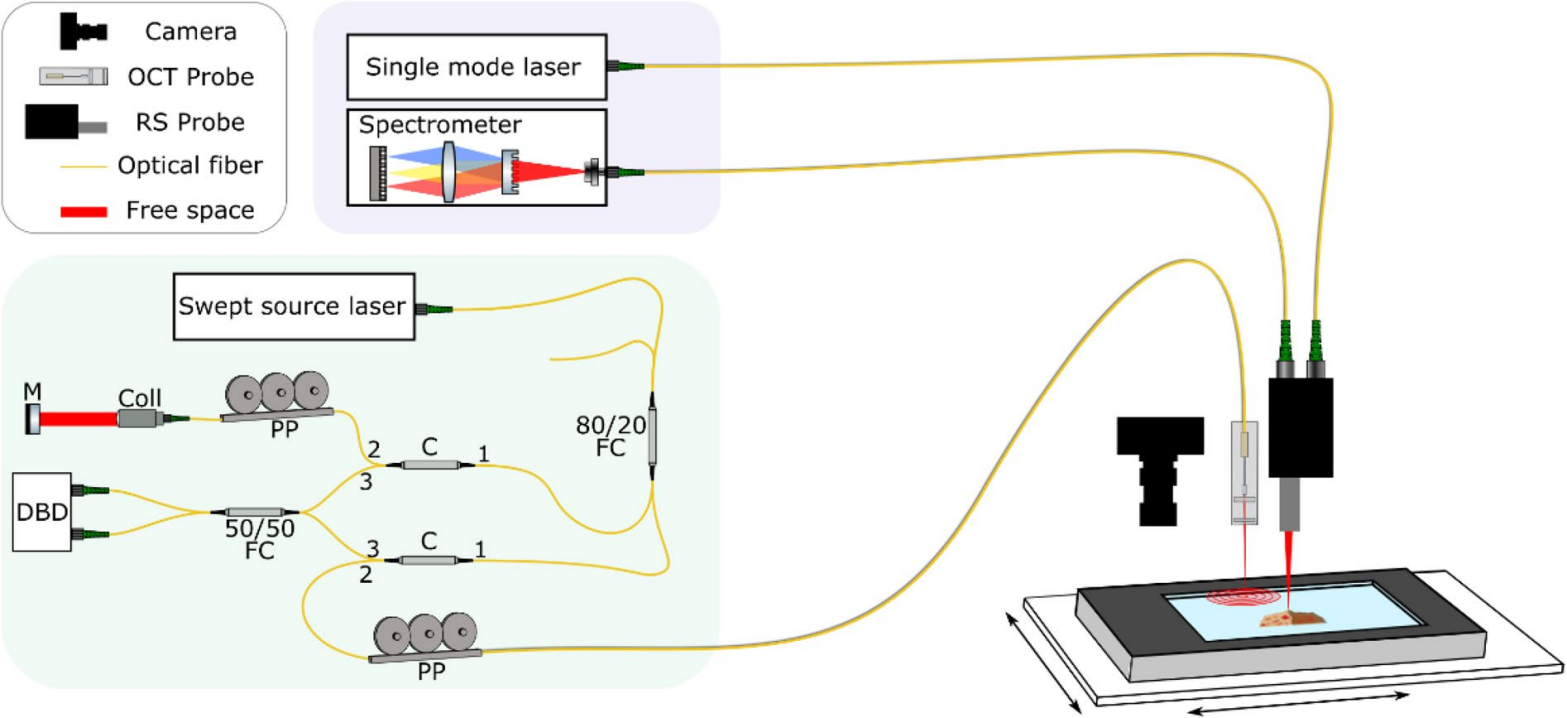
The morpho-chemical imaging system based on a combination of a forward-looking Raman probe and OCT probe. The Raman setup is built around a 785 nm single mode excitation source that is fiber-coupled to the Raman probe, which is mounted in parallel to the imaging OCT probe and focuses the excitation light onto the sample. The generated Raman signal is collected and guided to the spectrometer. The swept source OCT system is built around an akinetic swept-source laser, with a central wavelength of 1304 nm, a bandwidth of ~ 90 nm and a sweep frequency of ~ 173 kHz, which is connected through an 80:20 fiber optical coupler (FC) and a circulator (C) to the piezo-tube based scanning OCT probe. The setup includes the interferometric optical setup in Mach–Zehnder configuration with a dual balanced detector (DBD), polarization paddles for polarization management (PP) and driving electronics for the endoscope and a PC. The sample is observed by a brightfield camera and the region of interest can be selected by the user. Both systems are connected by an Arduino board, which is used to switch between Start-Stop the stage translation and data acquisition. M: Mirror, Coll: Collimator.

A particular optical modality provides information due to a specific contrast mechanism. This contrast can, however, have a multitude of origins, resulting in challenges when interpreting the data. Frequently, especially in OCT and RS, the interpretation of data is based on the experience of the user or a reference analysis, such as a histopathological examination of a biopsy sample. The combination of two complementary modalities has mostly been used to increase the diagnostic value of the underlying pathology but could also bear the potential to better understand and reference the fundamental origin of the signals. For example, while the origin for OCT and the Raman signals is based on different physical principles, i.e. OCT on scattering properties of the tissue and RS on the molecular composition of the tissue, the scattering properties of the tissue also depend on the molecular composition, making the origins of both modalities inherently connected. To investigate this relation, an optical setup was developed, based on two forward-viewing fiber optical probes, which allows consecutive acquisition of Raman maps and OCT volume stacks from large biopsy samples, Fig. 1. For more details on the system, see Materials and Methods section. Human urinary bladder biopsies were used as surrogate for those experiments.

**Figure 2:**
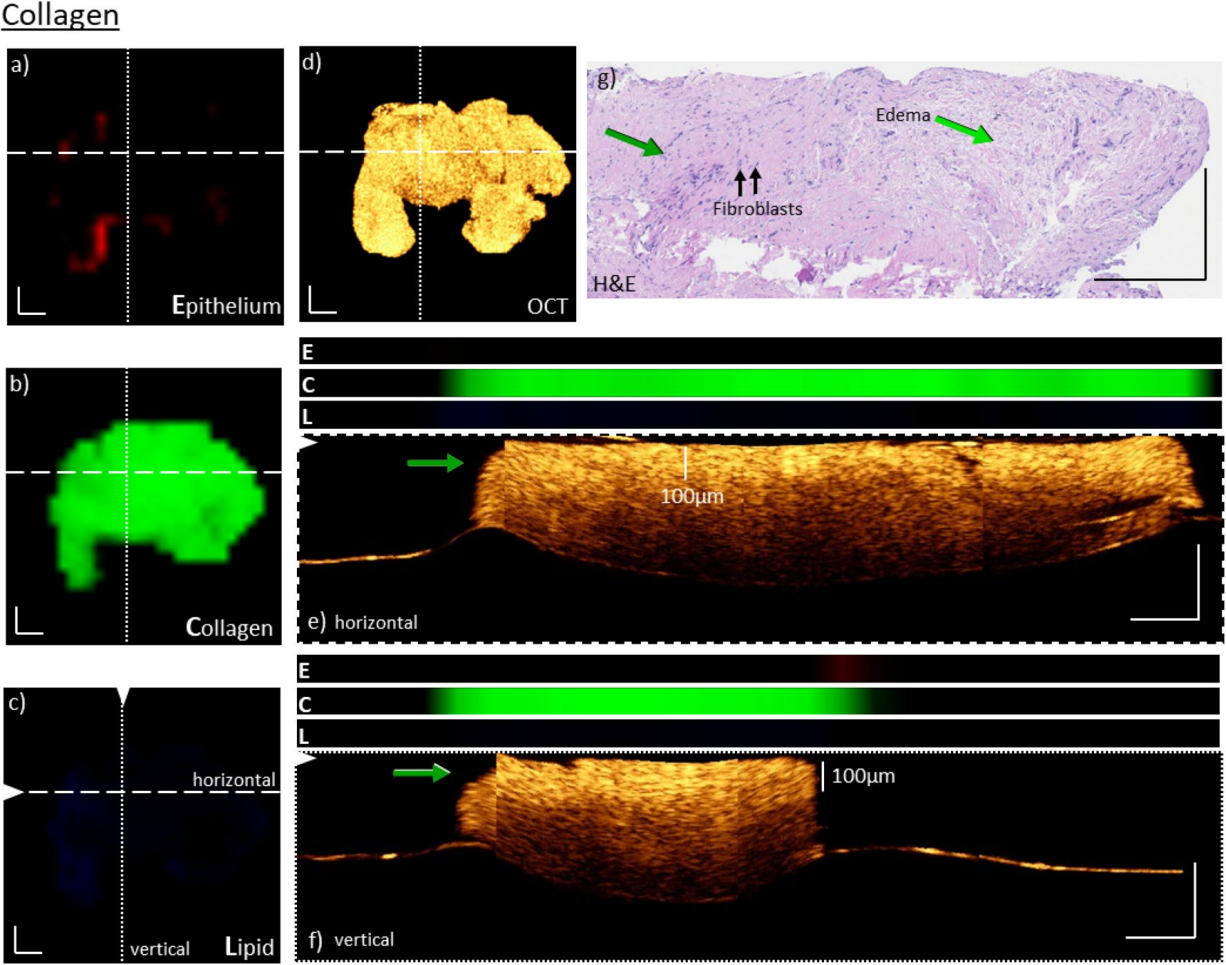
Co-registered morpho-molecular information of OCT and Raman images on a collagen rich bladder biopsy. Molecular Raman enface maps for epithelial (**a**), collagen (**b**) and lipid (**c**) content and enface OCT maximum intensity projection of ten slices from a depth of 120–150 µm (**d**). The Raman maps indicate a dominant and nearly homogenous collagen signal over the entire biopsy (**b**), whereas the lipid content and epithelial signal is not very pronounced (**a**,**c**). The molecular information corresponds well to the very bright OCT signal. Maximum intensity projections of 10 cross-sectional OCT B-scans are indicated for positions in the biopsy by dashed and dotted lines in the enface image (**e**,**f**), respectively. The co-registered molecular information for the three components (E: Epithelium, C: Collagen, L: Lipid) are plotted above. It can be seen that neither B-scans in (**e**) nor (**f**) do exhibit an epithelium layer, however, show features with a very bright appearance. For comparison, an H&E image of the respective biopsy is displayed in (**g**). In the H&E slice (**g**), only lamina propria tissue is present, green arrows. The whole section does not exhibit any epithelial cell layer. A region of edema within the lamina propria is identified. Further, two representative fibroblasts, building up the extracellular matrix constructed mainly of collagen fibers are pinpointed. Scale bars: 250 µm.

**Figure 3:**
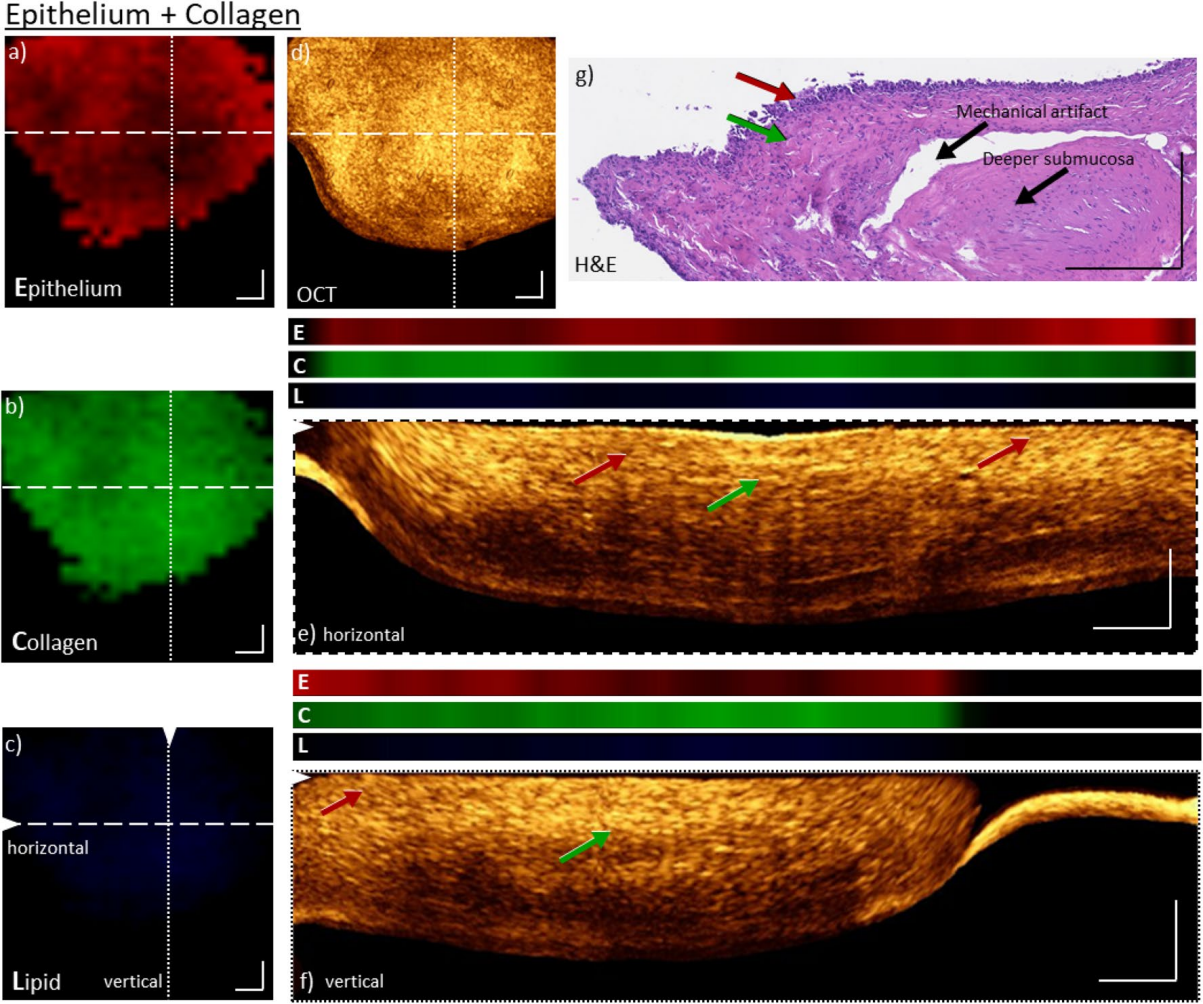
Co-registered, morpho-molecular information of OCT and Raman maps on a bladder biopsy with mixed epithelium and collagen signals. Enface Raman map indicates homogeneous distribution of epithelial (**a**) and collagen (**b**) signals, but no significant lipid contributions (**c**). The collagen signals are reduced in locations where the epithelial signal is increased and vice versa. For comparison, the enface OCT maximum intensity projection of ten slices from a depth of 90–120 µm is shown (**d**). The higher signal intensity in the OCT correlates quite well with the higher intensity in collagen signal (**b**). While a reduced intensity correlates better with the increase of epithelium tissue. The maximum intensity projections of ten cross-sectional OCT B-scans, from positions indicated by dashed and dotted lines in the images, are plotted in (**e**,**f**). The co-registered molecular Raman information (E: Epithelium, C: Collagen, L: Lipid) is plotted above, and shows a good overlap between the signals, i.e. brighter OCT signal overlapping with the collagen signals, while the darker areas correlate to the epithelial signals. For comparison, H&E image (**g**) of the same biopsy shows both collagen and epithelium regions, indicated by red and green arrows corresponding to epithelium and collagen Raman signals respectively. The epithelium is indicated with a red arrow. The green arrow is indicating the lamina propria with high collagen content. A mechanical artifact and the deeper lamina propria layer of the bladder wall are additionally visible. Scale bars: 250 µm.

**Figure 4:**
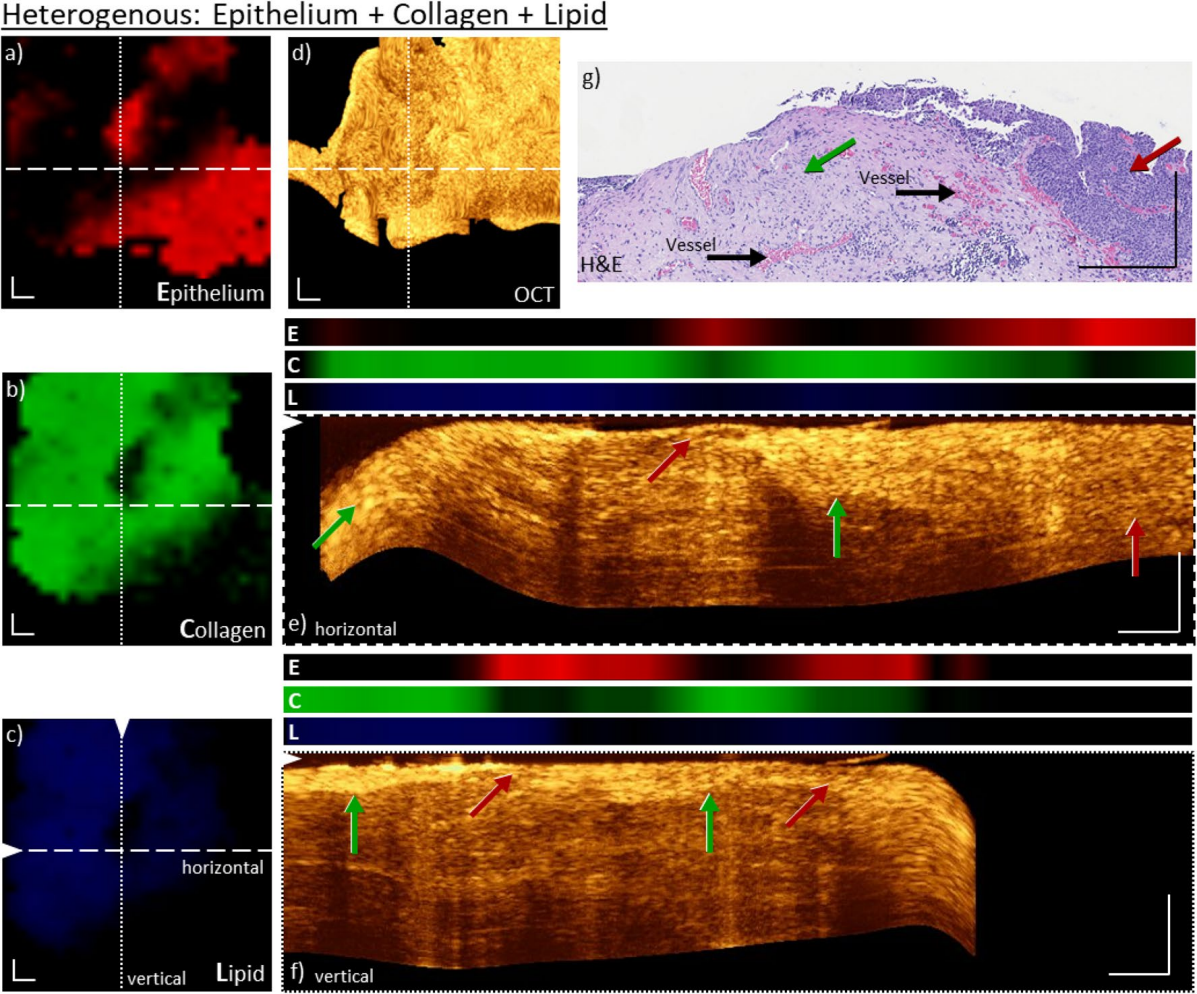
Co-registered, morpho-molecular information of OCT and Raman images on a bladder biopsy with strong epithelium and collagen signals. Enface Raman map for epithelium (**a**) and collagen (**b**) display primarily strong and heterogenous signals. Minor lipid signal is also present in the image (**c**). The corresponding enface OCT maximum intensity projection of ten slices from a depth of 120–150 µm is shown in (**d**). Maximum intensity projections of ten cross-sectional OCT B-scans, positions indicated by dashed and dotted lines in the enface image (**e**, **f**) with co-registered molecular Raman information (E: Epithelium, C: Collagen, L: Lipid). Both B-scans exhibit transition zones of bright and dark layers, which correlate to the Raman signals of collagen and epithelium (red and green arrows). Especially on the right-hand side of (**e**), the signal of the whole cross-section appears dark in comparison to the collagen signal next to it on the left. The same transition of epithelium and collagen is also visible in the Raman signals. For comparison H&E image of the respective biopsy (**g**) shows thickened epithelium (red arrow) and collagen rich lamina propria (green arrow). Vessels are located in the upper lamina propria layer of the bladder wall and indicated in (**g**). Scale bars: 250 µm.

**Figure 5:**
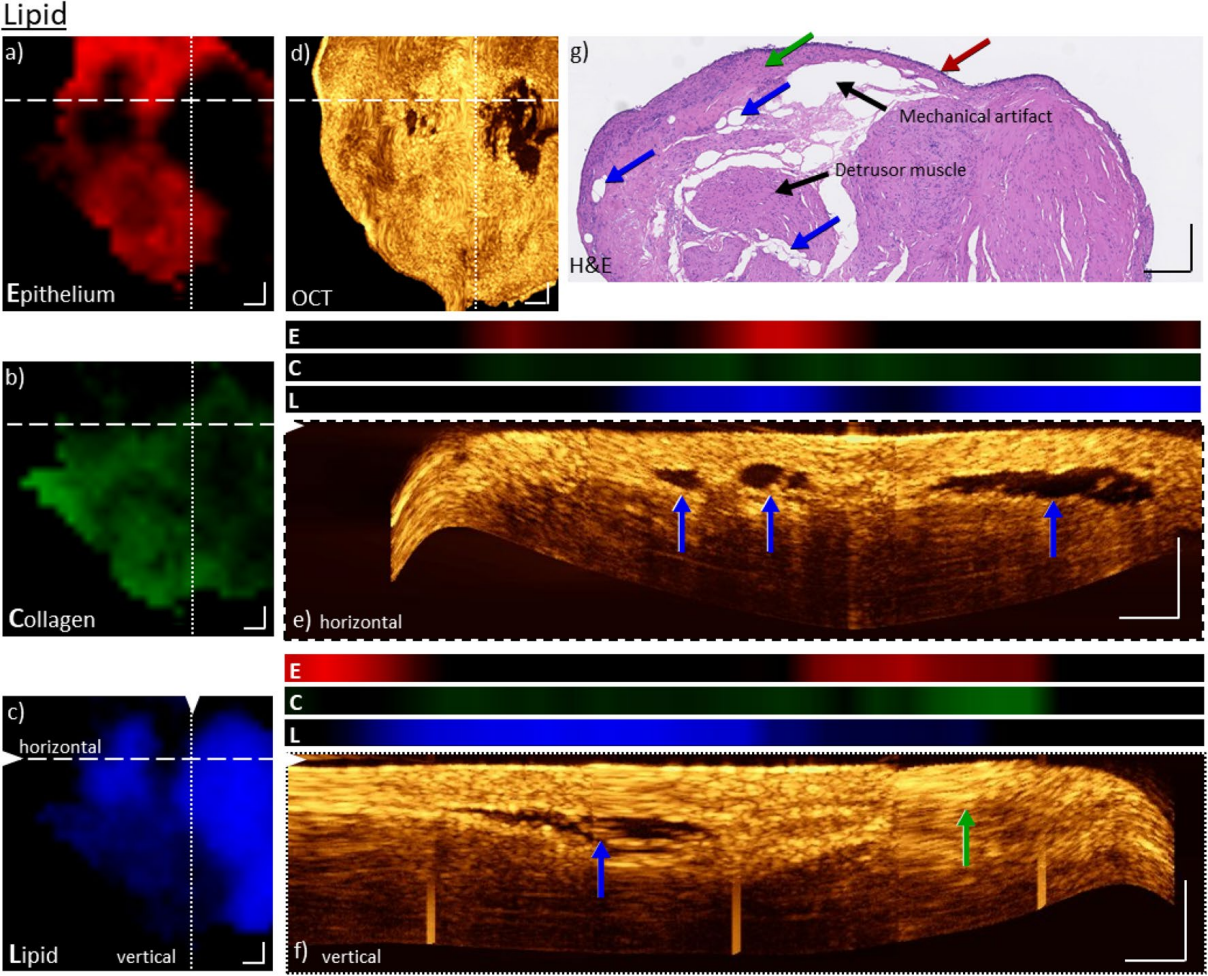
Co-registered, morpho-molecular information of OCT and Raman images on a bladder biopsy with high lipid contributions. Enface Raman maps for epithelium (**a**), collagen (**b**) and lipid (**c**), show the presence and distribution of all three components, and specifically very pronounced lipid pools. The enface OCT maximum intensity projection of ten slices from a depth of 150–180 µm (**d**) indicates black voids, correlating to the same locations as the lipid signals observed in the Raman map (**c**). Maximum intensity projections of ten cross-sectional OCT B-scans, positions indicated by dashed and dotted lines in the enface image (**e**, **f**) with co-registered molecular Raman information (E: Epithelium, C: Collagen, L: Lipid). In both images, the overlap of lipid Raman signals and the black voids in the OCT cross-sections in a depth of ~ 200 µm is visible. The straight vertical line arising from the bottom to the top in (**f**) is caused by artifacts of the used OCT probe. For comparison, the H&E image of the biopsy, where the blue arrows indicate the identified lipid pools, is shown in (**g**). Here, epithelium (red arrow), lamina propria (green arrow), detrusor muscle and mechanical artifact are also visible. Further, the collagen signal is correlated to a brighter area in the OCT cross-section (f, green arrow). Scale bars: 250 µm.

A total of 119 biopsies were imaged in a co-registered way with a forward-viewing endoscope-based setup. Because the imaging data for both modalities on biopsies was co-registered, it offers the opportunity to characterize the signal origins for both modalities and qualitatively correlate the data. To demonstrate a potential relation between both modalities the data was divided in separate groups, based on the information present in the Raman data. Depending on the biopsy the molecular signatures of the Raman signal show the presence of certain macromolecules commonly found in tissues. We have therefore divided the biopsies in three groups: lipid-rich, collagen-rich, and epithelial rich samples. For the manuscript a subset of representative biopsies was selected and analyzed. Figures 2, 3 and 5 show healthy bladder tissue, while Fig. 4 is based on data from an early tumor stage. Additionally, a comparison to histopathological data for those biopsies was also performed.

Figure 2 displays the co-localized morphological and molecular data for a representative biopsy, exhibiting very strong collagen signal, but only little epithelium and lipid signal in the Raman spectra. Additionally, the histological (H&E) information for the biopsy is presented. As described in the material and methods section, the individual Raman spectra were fitted by three components, representing spectral information of epithelium, lipid, and collagen, and are displayed as distribution enface maps of those components over the sample. For a better differentiation the epithelium was color-coded red (Fig. 2a), collagen green (Fig. 2b) and lipid blue (Fig. 2c). The strongest contribution appears in collagen, which is homogenous throughout the biopsy. The lipid and epithelium signals, on the other hand, are barely visible. Additionally, to the Raman maps, an OCT enface image, based on the maximum intensity projections of ten slices from a depth of 120–150 µm is also displayed, showing very bright signal features throughout the entire area of the biopsy, Fig. 2d. As reported by Garcia et al.^34^, collagen content in investigated tissue results in a bright OCT signal. To gain a better understanding of the underlying depth information of the data, B-scans are displayed, Fig. 2e,f, supporting the overall bright appearance of the signal in the top layer of the tissue. The collagen-rich layer has an approximate thickness of 100 µm, as indicated in the image. No other notable features are apparent in the B-scans. For comparison, the Raman signal for the same cross-section for each molecular component was plotted above the B-scan. Here again, the Raman signal and the OCT data correspond quite well, displaying strong features, where the scattering in OCT is also highest. The Raman signals are detectable in depth up to ~ 300 µm, while the OCT probe provides an efficient collection of signals within 950 µm. To support the morphological and molecular co-localized data, an H&E-stained section from the same biopsy is presented, Fig. 2g. The H&E slide of the biopsy confirms that the tissue is primarily composed of lamina propria, with no apparent presence of epithelium, confirming the observations from RS and OCT. It is possible that the epithelium was detached during handling of the biopsy after resection. The H&E slice does, however, indicate edema in one part of the lamina propria and two representative fibroblasts, having the task of building up the extracellular matrix mainly composed of collagen fibers, are indicated. These results support the notion that at least for collagen-related signals both modalities provide relatable information, and Raman spectroscopy could be used to specify the presence of collagen in the OCT data. This particular information could be a potential biomarker and define the transition from benign to malignant tissue.

Clearly, the sole presence of collagen features is undeniably a special case for this sample and in most biopsies more than one component can be found. Figure 3a,b present an example were the enface Raman maps not only display collagen features, but also signal contributions from epithelium tissue. Both molecular maps show that the constituents are distributed homogeneously throughout the biopsy, except for the central location where the collagen density is increased, while the epithelium contribution is reduced. Again, no lipid signal can be observed in the Raman map, Fig. 3c. The corresponding, enface image of OCT is displayed in Fig. 3d. Because the data was normalized over the entire data set for both modalities it is also possible to compare the observations between the different samples (Figs. 2, 3, 4, 5). Hence, taking into consideration the observation from Fig. 2 i.e. a high collagen presence resulted in a very bright OCT signal, here, one should see overall an apparent reduction in the OCT signal intensity, except in the location where the collagen Raman map shows higher signals. Indeed, the OCT enface image Fig. 3d has a reduction in brightness when compared to the OCT enface image Fig. 1d. More specifically, the signal is not homogenous throughout the entire sample, but has a central location where the OCT signal exhibits a particular high brightness, while the other positions have considerably lower intensities. When comparing the OCT enface projection to the Raman maps of the same locations that display a strong OCT signal, an increase in collagen contributions in the Raman image can be seen. The reduced OCT signal, on the other hand, correlates with a reduction in collagen, but an increase in epithelium signal in RS. To better comprehend the local morphological differences in depth a cross-sectional representations for the indicated positions of the enface OCT are displayed in Fig. 3e,f. Additionally, the molecular signals of the epithelium, collagen and lipid from Raman spectroscopy for that particular cross-section are displayed above the image. Here again, it is possible to relate the OCT depth profiles, which show a heterogeneous variation of the signal intensity, to the molecular variation from the enface Raman maps for the presence of collagen and epithelium tissue. It can be seen that the signals from both modalities co-localize extremely well, indicating that the pre-processing and the co-registration worked appropriately. Regions of more collagen (lamina propria) are giving a brighter OCT signal in depths of 100—200 µm, which was also previously observed^34^, while regions where the OCT signal is reduced a stronger epithelium signal in Raman is visible. To support this information, an H&E slice for this biopsy is presented, Fig. 3g. The bladder wall with an epithelial layer and lamina propria can be seen, confirming the observations provided by the cross-linked OCT and RS information. Furthermore, mechanical artifacts and the deeper layer of the lamina propria are visible.

An additional example for a mixed epithelium and collagen sample is shown in Fig. 4. Here, a similar relation between the decomposed Raman signals and the OCT signal, but in a more heterogeneously way, as described in Fig. 3, is present. For example, the B-scans unveil transition zones of lamina propria to epithelium, Fig. 4e,f, and the co-localized molecular signals from the RS also indicate such transition zones. The collagen rich layer is extending in depth from ~ 0–50 µm up to ~ 0–250 µm, Fig. 4f,e, respectively. On the right side of Fig. 4e, the OCT signal exhibits a homogenous intensity over the entire depth and the molecular contributions to the Raman signal are mainly from the epithelium tissue. The histopathological image, Fig. 4g, shows thickened epithelium on the right-hand side and lamina propria on the left-hand side. This may support the finding in Fig. 4e of the thick epithelium layer and its homogenous appearance. Additionally, vessels are indicated in Fig. 4g.

There are also cases where other signal features can occur in both modalities. Figure 5 shows a representative biopsy with more heterogenous behavior, i.e. where besides the collagen and epithelium signals also substantial contributions of lipids are present, Fig. 5a–c. In comparison to previous cases, Figs. 2, 3 and 4, the amount of lipid signals is substantial and well defined, and results in a decrease in epithelium and collagen signals in those particular locations. As was previously shown, the signal intensity/brightness corresponds well to the scattering property of the tissue, i.e. collagen contributions resulted in a high OCT signal, while epithelium tissue resulted in a reduced brightness in comparison to collagen, Figs. 2, 3 and 4. Taking into consideration that lipid accumulations are highly homogeneous and low scattering^37^, the observed OCT signal should be even more reduced, as in comparison to epithelium tissue. Figure 5d shows the corresponding enface OCT image, which has next to the readily observed variation of signal intensity attributed to variation of epithelium and collagen signals, very apparent and distinct black voids. Taking only the OCT information into account, those voids could also easily be assigned to mechanical rupture of the tissue, which frequently occurs through stress during the handling. However, by taking the information extracted from the Raman data those voids can be clearly related to accumulations of esterified lipids. The present lipid signal (Fig. 5c) is significantly higher than present in the previous biopsies in Figs. 2, 3 and 4 and overlaps with the region of the black features in the OCT image. Based on the depth information of OCT, the lipid signals are co-localized with the black voids in the OCT B-scans in depth (Fig. 5e,f). RS is giving evidence, that those black voids are not caused by mechanical forces but are filled with esterified lipids. Furthermore, the beneficial depth information of OCT enables the detection of the RS lipid intense signal regions in a depth of ~ 100—250 µm. Additionally, the RS collagen intense signals are co-localized with the bright OCT signals at a depth of ~ 0 to 50 µm. Through the morpho-molecular cross-linking of OCT and RS, the signal origin and the understanding of the present biological tissue is substantially enhanced. Additionally, the H&E slice support the presence of lipid pools within the bladder wall and also epithelium and lamina propria, Fig. 4g. Besides mechanical artifacts, the detrusor muscle is visible. Those results strongly support the notion that, while the signal origin of both modalities is complementary, they both provide a very beneficial information that assists to explain the corresponding modality.

## Discussion

In this work we presented a qualitative correlation between morphological information established by OCT with the molecular information provided by RS. The results strongly indicate that despite the different contrast origins of both complementary modalities the information is intrinsically relatable and can provide a more comprehensive characterization of the investigated tissue. The presented results support the notion that OCT can be used as a pre-screening technology, rapidly providing information on morphological structures and identifies abnormalities, while Raman spectroscopy can be used to provide adjunct molecular specific information from those locations, enabling a molecular profiling of the OCT signal origin. The combined and complementary information enables a better understanding of the underlying structures for the detected signals, strengthening the prospective for clinical use of multimodal imaging and spectroscopy devices.

The co-localization of the modalities and the imaging information was achieved by implementing a multimodal optical setup based on two front-viewing fiber optical probes and applied to the imaging of bladder tumor biopsies. The co-registered morphological and molecular information was summarized on four representative cases (3 healthy normal tissue/1 early tumor) and qualitatively compared between the modalities and the corresponding histopathological ground truth. Due to the access to the histopathological gold standard information, it was not only possible to relate the molecular information between OCT and RS, but also to provide a proof for the observations. The determined molecular signatures for epithelium, collagen, and esterified lipids were directly relatable to observations in OCT tomograms, which lack the specificity and require additional confirmation by H&E histology.

The observations of the present work show that strong scattering contributions in OCT tomograms in bladder biopsies typically correspond to collagen from the connective tissue layer called lamina propria. The presence was observable in most OCT tomograms from a large number of samples, varying in different degrees. However, because the signal is not specific, the signal generating structures cannot be safely deduced by OCT alone. Collagen has, however, a highly unique Raman signature, and can be differentiated from other protein signals. As such, the mapping of the Raman signal of collagen accurately provides the distribution of collagen across the sample. It is possible to extract ‘pure’ component spectra, which closely represent true and pure molecular constituents of a sample, from the Raman data. Here, we used a combination of vertex component analysis (VCA), which extracts endmember spectra from a data set, followed by multivariate curve resolution (MCR), which can further optimize the endmember, providing nearly pure components, Fig. 8. Mapping the signal of the components provides information on the distribution of the molecular constituents in the sample. Moreover, by performing the measurements in a co-localized fashion, it was possible to qualitatively correlate OCT and Raman information, showing that the bright signal features in OCT do correlate to an increase of collagen in the tissue. The individual OCT images and Raman maps were normalized to the highest signal intensity over the entire data set of the present biopsies, allowing a signal comparison of the OCT and RS data between the individual biopsy. It could be shown that the variance in the OCT signal intensity is also correlated to the variation in the Raman signal intensity. Besides a very distinct collagen signal, Raman signal of esterified lipids has a very unique Raman signature, and additionally, have a very high scattering cross-section, which makes them easily detectable. In OCT tomograms the signals from esterified lipid depositions appear as very unspecific black voids, which could easily be due to a presence of vasculature or a mechanical rapture of the tissue. Here again, the combination of OCT-RS provides a picture of the morphological information and the related molecular information from the sample. In fact, Raman spectroscopy is also capable to differentiate intracellular compartments, such as nuclei, cytosol, intracellular vesicles, etc.^10,40^, however, due to the focal spot size of approx. 100 µm, and a sampling depth of ~ 300 µm, only an average information from the tissue was acquired. Nevertheless, as shown in previous work, the average signal from epithelial tissue has also a quite distinct Raman signature, exhibiting mostly integrated signals of cytosol, nuclei acid and membrane lipids^41^. Using the aforementioned data-decomposition approach it was also possible to extract a signal that presents the well-known epithelium Raman spectrum. This signal was also mapped with the two other components and compared to the OCT tomograms. In OCT, the presence of epithelium tissue results in a reduced scattering, when compared to the signals from collagen, and as expected, is generated in the most top layer of the sample, followed by the signal from a stronger scatterer. Here again, the signals match very well with the Raman maps of epithelium tissue, Figs. 3 and 4. The OCT data contrast is not strongly influenced by the focus position, since the large confocal parameter for OCT (~ 950 µm) ensures similar illuminance along depth. Additionally, sufficient detection efficiency of Raman photons is ensured within the respective confocal depth of approx. 300 µm. This example focuses on the overall comparability and understanding of different signal strength for morphological and molecular components. The presented analysis strengthens the evidence that the signal origin for both modalities is inherently linked and can be used for an in-depth understanding about the sample composition.

The physical connection of both signal origins is linked by the relation of the scattering properties of a tissue with the molecular composition, influencing the signals of both modalities. The combination of both complementary modalities not only provides an improved diagnostic value but can also be used to aid and reference the observations of the other method, i.e. while conventional RS provides no information on the depth, it unveils the molecular signature of the tissue, allowing to reference the observations in OCT. On contrary, OCT is not very specific, but provides a depth perception to the origin of the RS signal. The analysis establishes for the first time that two complementary imaging modalities are providing a relatable assistance to each other and support a clearer clinical picture of investigated tissue composition and health. This new path could lead to more sophisticated devices for visual and machine aided analysis of tissue being faster, label-free and accessible for clinical use. More importantly, the presented results open a new way of understanding and cross-linking well established imaging technologies, marking a further step towards fusing two complementary modalities to one single system. Additionally, in future implementations, such as fiber-probe based combination of OCT with RS, the addition of spatially offset Raman spectroscopy (SORS), as outlined by Chen and Dholakia^42^, could be of significant interest, as this allows to relate the Raman-information to the observable depth information.

## Materials and methods

The OCT and RS data were acquired during a study, approved by the Ethical Committee at the Capital Region of Denmark, H-17015549, and complied with the guidelines of the 2013 Declaration of Helsinki. Furthermore, a data processor agreement between the universities in Jena and Vienna and the Capital Region of Denmark was established (HGH-2018-038. I-suite nr. 6639). Prior to the operation, each patient was informed and gave written and informed consent to have samples for the study taken. In total 119 biopsies from 44 different patients were resected during bladder tumor examination, 66 of which were non-tumor, 3 PUNLMP (papillary urothelial neoplasm of low malignant potential), possessing a limited probability of cancer progression^43^, 1 carcinoma in situ and 49 were cancerous biopsies. Out of the 49 cancerous samples, 48 were staged pTa and 1 pT1a, 12 of which were high and 38 were low grade.

The morpho-molecular imaging was performed on a combined, forward-viewing probe based automated system in a co-registered manner. Detailed description on the biopsy handling, the multimodal imaging setup, data acquisition procedure and data analysis has already been described by the authors^3^, and will be only briefly summarized. The OCT and RS data was acquired subsequently after choosing a region of interest (ROI) with an acquisition time of 1 min for 9 OCT stacks and 13 min for 900 Raman-point measurements in the same region on a ~ 4 mm² sample. The OCT and Raman probe were mounted on an in-house designed and build holder, with a fixed offset position between the two probes, enabling co-registered data acquisition. The OCT setup and data acquisition workflow is as follows: The OCT setup incorporated an akinetic swept source laser (Insight Photonic Solutions, Inc., Lafayette, Colorado) with a central wavelength of 1304 nm, a bandwidth of ~ 90 nm and a sweep frequency of ~ 173 kHz. The interferometer is in Mach–Zehnder configuration (Fig. 1). The power at the tip of the OCT endoscope was 11 mW with a measured axial and lateral resolution of 12 µm and 28 µm in air, respectively. The confocal parameter was ~ 950 µm. Prior to the measurement a calibration of the piezoelectric tube driven scanning probe was performed. For reconstructing volumetric OCT stacks, the scanning pattern was imaged on a position sensitive device for creating a look up table (LUT). This LUT in combination with an algorithm enabled the remapping of the spiral scan onto a square-cartesian grid leading to a cylindrical 3D-volume with a size of 500 × 500 × 220 pixels. The pixel size in depth after the Fourier transformation was ~ 4 µm, resulting in ~ 3 µm in tissue considering a tissue refractive index of 1.33. The scanned volume, depending on the diameter of the spiral scan, ranged from approximately 0.7 mm^3^ (1 mm^2^ × 0.7 mm) up to 1.4 mm^3^ (1.96 mm × 0.7 mm). The acquisition rate for one volume was 0.5 Hz. The remapped 3D volume consisted of 510 consecutive circles (B-scans) with 340 A-scans each. Since one spiral scan was not enough for imaging most of the biopsies (size between 1 and 15 mm^2^), subsequent raster OCT volume acquisitions were performed. Stitching, aligning and rotating of those OCT volumes led to a coverage of the whole biopsy.

Following the acquisition of the OCT stacks the system switched automatically to the Raman side, and the acquisition of the hyperspectral Raman image was performed. Because both imaging probes were mounted on a common holder, the probe-to-probe distance was fixed by design, ensuring that identical ROIs were sampled. The Raman signal was collected by an in-house developed Raman fiber optic probe, equipped with 105 µm multimodal fiber that was connected to a 785 nm single-mode excitation laser (Fergy-Laser, Princeton Instruments). The probe was designed to image the exit aperture of the optical fiber with a magnification of 1 onto the sample plane, resulting in a nominal spot size for the Raman acquisition of approx. 105 µm. For all acquisitions 900 points distributed in a rectangular pattern (30 × 30 points) equally spaced across the biopsy were acquired. The generated signal was collected by the probe and imaged with a two-fold magnification onto a collection fiber with a diameter of 200 µm. Depending on the specific sample, signals can be collected from depths of up to ~ 300 µm. The fiber was connected to a spectrometer (Acton Series LS785, Princeton Instruments), which was equipped with a back-illuminated deep-depletion charge-coupled device (PIXIS 100BR_eXcelon, Princeton Instruments) with a 1340 × 100 imaging array and 20 × 20 μm sized pixels. The Raman measurements were performed with an output power of 70 mW at the distal end of the fiber probe, which was focused to a spot size of approx. 100 µm and with a spectral resolution of 5 cm^−1^. The signal covered a spectral region from 370 to 3600 cm^−1^. For the processing only the region between 600 and 3200 cm^−1^ was used. The acquisition time for each spectrum was 1 s.

## Data pre-processing and analysis strategy

There are certain challenges to combine the two distinct modalities. Firstly, the OCT data has to be flattened to account for the normalization procedure of the Raman data. Secondly, correcting for artefacts outside of the biopsy. Thirdly, the images are acquired spatially offset to each other, and have to be properly overlapped. Finally, the images have to be properly scaled, since the x–y dimension of OCT is on the order of 700 × 700 pixel, while the Raman image data is only 30 × 30 pixels. In the following we will describe and outline the established approach to process and correlate the data.

**Figure 6:**
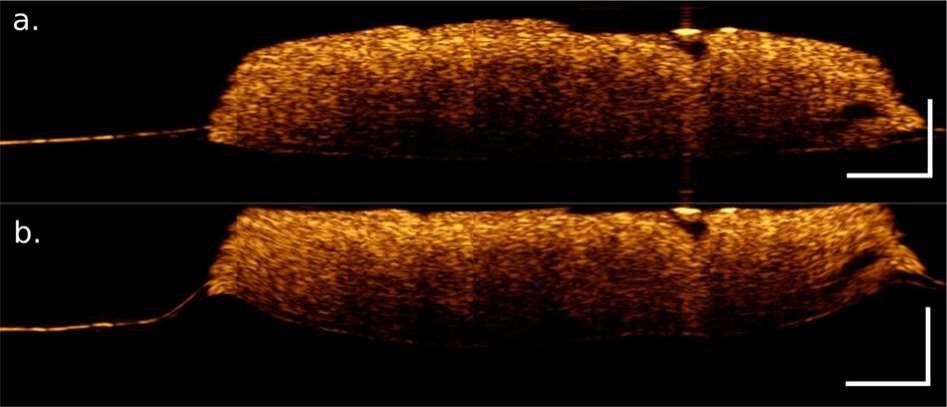
Surface adjusted OCT image (averaged over 5 B-scans) and extracted pure component spectra from the biopsies. Most of the biopsy samples have an uneven surface, resulting in challenges during the analysis, (**a**) To remove the curvature observed in the OCT images an inhouse developed algorithm, based on gradient surface position detection was used to determine the surface location and adjust, i.e. flattening, the surface, (**b**).

To address the curvature of the OCT data, an in-house developed algorithm was implemented. The approach for finding the leading edge of the surface of the biopsy, i.e. the surface, is achieved by finding the first maxima of a derivative function of a smoothed representation of the individual A-scans, see Fig. 6a,b. By shifting the A-scan vector to zero and applying this to the entire biopsy creates the flattening of the surface. To correct for the scattering artefacts outside of the biopsy each individual curvature corrected A-scan was integrated, resulting in an integrated signal map. By applying an automated thresholding, based on minimizing the Euclidean distance a binary mask was created, which was applied to the stack to remove artefacts located outside the biopsy. In case residual artefacts remained, removal was performed manually. The masked and integrated signal map was used for the correlation of the OCT data and the Raman data. Before the correlation the Raman data had also to be conditioned, i.e. after the aforementioned data pre-processing of the Raman data the signal of the high-wavenumber region was integrated to create a mask. It is important to use the integrated signal, because solely relying on a single band could result in a mask that only represents the distribution of a particular macromolecule in the sample, while the integration over the high-wavenumber region captures the total molecular distribution quite well. To perform the image registration the Raman image was first resized to closely match the size of the OCT image. The resized Raman image and mean projection of the OCT stack were transformed to phase-correlated polar coordinates^44^. The scale and the translation coordinates were used on the subsequent Raman images to achieve a good overlap. One has to keep in mind that due to the largely different image sizes and the different signal origins an overlap was not always perfectly achieved.

**Figure 7:**
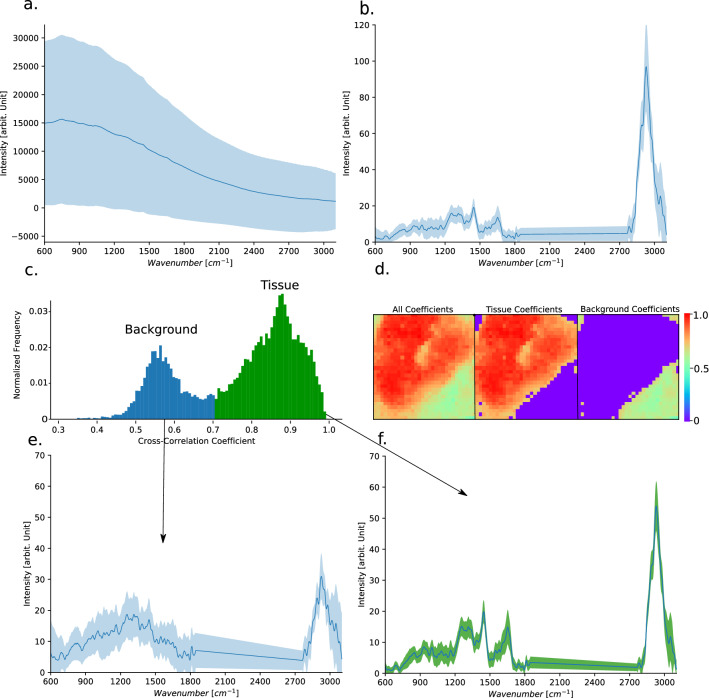
Raman spectra of biological tissue, specifically of bladder, contain significant autofluorescence contributions and require substantial data-pre-processing. The mean raw-spectrum and the standard deviation is displayed for all bladder biopsies, (**a**) The background-corrected and area-normalized mean-spectra with standard deviation are shown in (**b**) Normalized correlation coefficient-value occurrence plotted as a normalized histogram, revealing two separate distributions, i.e. background and tissue below and above a correlation-value of approx. 0.7 (**c**) An example for the separation of background signal and tissue signal, a map of the correlation values is presented for an entire biopsy and the separation, based on the presented threshold value of 0.7, (**d**) Additionally, the mean and standard deviation spectra are plotted, i.e. for correlation-values < 0.7 (background), (**e**) and correlation values > 0.7 (tissue), (**f**).

The imaging data of both modalities are inherently multidimensional. The individual A-scan of an OCT image provides a depth profile, based on the scattering property of the tissue, containing up to 220 individual variables. The Raman signal of an imaging set, on the other hand, being a hyperspectral data set, is also inherently multivariant, as it encodes the molecular profile of the sample for each data point, containing up to 1340 interrelated variables. Besides, the raw Raman data also contain extensional background contributions from tissue autofluorescence and spectra, which do not belong to the biopsy. It is, therefore, indispensable to perform pre-processing before any dimensionality reduction of the Raman data. Biological samples, and specifically tissue, contain significant background contributions from tissue autofluorescence, Fig. 7a). To remove this autofluorescence signal the spectra are first corrected for dark-current and the constant offset bias of the CCD-detector, using recorded dark-spectra. Furthermore, the spectra are also corrected for cosmic spikes, using an in-house developed algorithm, based on a combination of differential peak detection and interpolation between adjacent pixel values. The resultant spectra are intensity calibrated with spectra acquired from a standardized white-light, National Institute of Standards and Technology (NIST) (Kaiser HCA calibration accessory), followed by a background correction, using the asymmetric-least squares (ALS) approach^45^. The resulting spectra are then area-normalized and presented as mean-spectrum with standard deviation as in Fig. 7b. Because the acquired maps also contain regions where no tissue is present, these signals can influence the data processing and it is, therefore, important to remove those regions. To achieve this a cross-correlation was performed between the mean-spectrum of the dataset and each individual spectrum. To properly visualize the results, the normalized coefficients are plotted in a normalized histogram, Fig. 7c. There are two distributions present: below 0.7, where the values correspond to the data not related to the tissue (background); and for values above 0.7, which correspond to tissue data. To have a better visual understanding of this information a map based on the correlation values is presented for a biopsy, where the coefficient values are displayed separately for the background and the tissue, Fig. 7d. It can be seen that the tissue and the background related values are quite well separated. Furthermore, the mean and standard deviation spectra are plotted for background and tissue areas, Fig. 7e,f, respectively. The presented pre-processed spectra were used for further data analysis.

**Figure 8:**
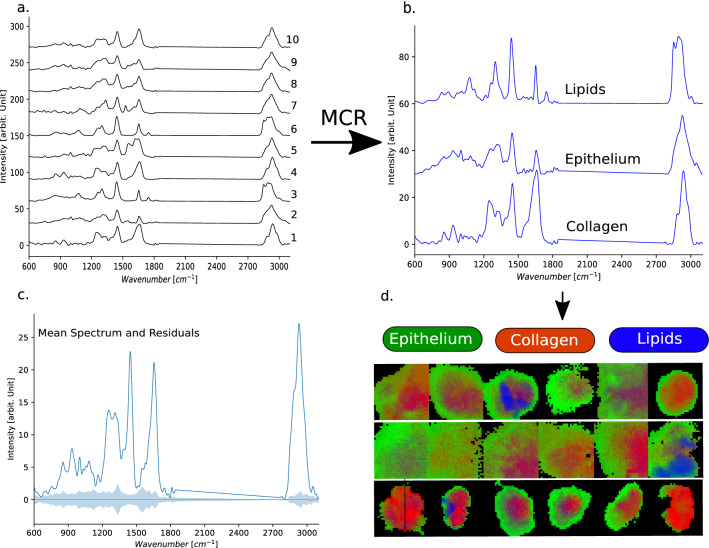
To determine ‘pure’ component spectra, which best represent the three major tissue components, i.e. epithelium tissue, collage, and esterified lipids, a combination of VCA and MCR-ALS was used. Ten endmember spectra extracted from the dataset, using vertex component analysis are displayed, (**a**) Three components from the VCA-endmembers were selected as the initial guess for MCR, i.e. Nr. 1 (collagen), Nr. 3 (esterified lipids), and Nr. 8 (epithelium tissue) and the resulting pure component spectra are shown, (**b**) The mean spectrum of the data set and the standard deviation of the residuals is plotted, (**c**) To better visualize the results the maps on the distribution of the three components are presented as RGB-maps for the several biopsies, (**d**).

To reduce the dimensionality of the Raman data, a combination of VCA and MCR-ALS was used to extract the endmember spectra, which represent the components for lipid, collagen, and epithelial tissue^40,46^. The spectra extracted through VCR usually differ from pure component spectra, as they can also contain contributions from other molecules. VCR only extracts spectra, which are present in the data set. It is especially challenging to determine any macromolecular variations for the cell-rich endothelial region, where the intrinsic composition of the cell is highly complex. Nevertheless, many of the macromolecules, e.g. collagen and esterified lipids, are present in very high concentrations in tissue, and are easier found in a large data set. When looking at spectra from cells (either from Raman-mapping experiments or sampling a very large number of cells), also contributions from nucleic acids will occur. Of course, this can differ when highly concentrated components, other than those mentioned, are present in the sample, e.g. bone, carotenoids, retinol, etc. However, because OCT is only sensitive to macroscopic differences, many potential contributions from small molecules in the Raman data, such as nucleic acids, carotenoids, etc. would not be suitable for a correlation to the OCT data. As such, we have focused on establishing components, which would also be assessable through OCT. VCA was used to determine ten endmember spectra and performed on the entire set of the presented biopsies. The resulting endmember components are shown in Fig. 8a. It can be seen that spectra have overlapping signatures, e.g. Nr. 1 and 4; Nr. 3 and 6; Nr. 2, 8, 9. Adding additional components will therefore not necessarily provide additional and relevant features. To further optimize the endmember spectra, MCR-ALS^47^, based on the implementation in Python by Camp Jr. was used^48^. In contrast to VCA, MCR is an iterative algorithm, which optimizes the concentration and the endmember spectra in an alternating least-squares way under constraints that provide chemically meaningful profiles through ALS. Moreover, it also accepts a guess for the pure spectral contributions. Here, we used the endmember spectra, Fig. 8a, Nr. 1 (collagen), Nr.3 (lipid), and Nr. 8 (epithelial tissue). Each of the selected endmembers have well defined spectral features, which we have also described previously, and which match the known signature of the components^2,49^. The resulting spectral profiles are presented in Fig. 8b. To assess the extracted pure components, the preprocessed spectra of the dataset were fit by the components and the mean spectrum and the standard deviation of the residuals is plotted in Fig. 8c. It can be seen that the residuals are quite low, in comparison to the entire information present in a typical spectrum. To further elucidate the results, the calculated concentration coefficients of the three components were plotted for a subset of biopsies and represent the spatial distribution of collagen, epithelium tissue and esterified lipid across the biopsies as RGB-images, where R(ed)—codes for collagen, G(reen)—codes epithelium tissue, and B(lue) – codes for esterified lipids, Fig. 8d. It can be seen that collagen and epithelium tissue are very dominant in the biopsies, whereas lipid contributions are only present in some biopsies. Note: because the biopsies have different sizes, scalebars were omitted from the maps for clarity.

## Data Availability

The imaging datasets generated and analyzed during the current study are available from the corresponding author on reasonable request.
